# A Novel Framework for Abnormal Risk Classification over Fetal Nuchal Translucency Using Adaptive Stochastic Gradient Descent Algorithm

**DOI:** 10.3390/diagnostics12112643

**Published:** 2022-10-31

**Authors:** Deepti Verma, Shweta Agrawal, Celestine Iwendi, Bhisham Sharma, Surbhi Bhatia, Shakila Basheer

**Affiliations:** 1 Department of Computer Application, SAGE University, Indore 452020, India; 2 Institute of Advance Computing, SAGE University, Indore 452020, India; 3 School of Creative Technologies, University of Bolton, Bolton BL3 5AB, UK; 4 Department of Computer Science & Engineering, School of Engineering and Technology, Chitkara University, Baddi 174103, India; 5 Department of Information Systems, College of Computer Science and Information Technology, King Faisal University, Al Ahsa 36362, Saudi Arabia; 6 Department of Information Systems, College of Computer and Information Science, Princess Nourah Bint Abdulrahman University, P.O. BOX 84428, Riyadh 11671, Saudi Arabia

**Keywords:** NT (nuchal translucency), fetal abnormality, Adaptive Stochastic Gradient Descent Algorithm (ASGDA), risk score evaluation

## Abstract

In most maternity hospitals, an ultrasound scan in the mid-trimester is now a standard element of antenatal care. More fetal abnormalities are being detected in scans as technology advances and ability improves. Fetal anomalies are developmental abnormalities in a fetus that arise during pregnancy, birth defects and congenital abnormalities are related terms. Fetal abnormalities have been commonly observed in industrialized countries over the previous few decades. Three out of every 1000 pregnant mothers suffer a fetal anomaly. This research work proposes an Adaptive Stochastic Gradient Descent Algorithm to evaluate the risk of fetal abnormality. Findings of this work suggest that proposed innovative method can successfully classify the anomalies linked with nuchal translucency thickening. Parameters such an accuracy, recall, precision, and F1-score are analyzed. The accuracy achieved through the suggested technique is 98.642.%.

## 1. Introduction

**Figure 1:**
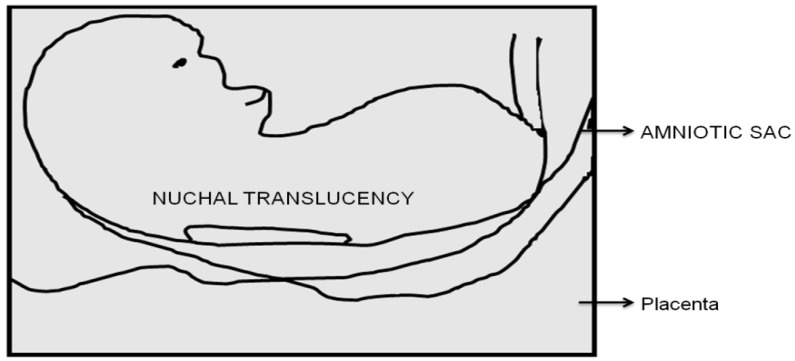
Identification of fetal nuchal translucency.

Child death is a critical issue that must be prioritized to rescue children. A child’s abnormality may be determined by using ultrasound technology to diagnose the prenatal condition within a certain time frame to provide better treatment. Prenatal ultrasound screening can detect many major fetal anomalies, and as a result, congenital flaws are regularly identified before birth. One in 700–800 newborns will be born with Down’s syndrome, and similar numbers will be born with other chromosomal and significant genetic diseases if prenatal screening is not implemented [1]. Because abnormal babies are more likely to miscarry than normal fetuses, the prevalence of abnormalities in early pregnancy is higher. Ultrasound scanning (US) is now universally accepted as the most important approach for detecting congenital abnormalities in the fetus during pregnancy. It detects the majority of prenatal abnormalities, but not all of them. Prenatal ultrasound imaging devices are used to diagnose Down’s syndrome, a genetic disease caused by an overabundance of a gene on chromosome 21. In terms of physical expression, mental growth retardation, and cognitive inability, Down’s syndrome has an impact on children’s health. It causes thyroid or heart illness. Early identification methods for Down’s syndrome make it possible to rescue children. Down’s syndrome children experience a variety of issues, including mental, physical, heart, ear, and vision impairments [2]. Figure 1 describes the identification of fetal nuchal translucency (NT). Fetal abnormalities are divided into two categories: structural and functional. The heart, lungs, kidneys, limbs, and facial features of a developing newborn are all affected by structural defects. Structure birth abnormalities include heart defects, missing toes, cleft tip, and spina bifida. A functional anomaly affects the way a body organ or system, such as the brain, nervous system, or sensory perception, functions. Blindness, seizures, impairments in development, Down’s syndrome, and muscular dystrophy are all examples of functional birth abnormalities. Some fetal malformations can influence the structure and function of the baby [3].

For a pregnant woman’s improved health care, accurate fetal parameter dimensions of US scans are critical. Predicting prenatal anomalies would make treatments more effective in lowering infant morbidity and death, which would benefit families, society, and the healthcare system. Fetal biometric measurements and nuchal translucency thickness are critical criteria in the identification of fetal abnormalities in obstetrics [4]. The biparietal diameter (BPD), gestational sac (G.Sac), abdominal circumference (AC), head circumference (HC), and length of the femur are among fetal biometric characteristics (FL). These biometric data are utilized to determine the fetus’ gestational age and identify aberrant growth trends. During the late first trimester and early second trimester (80 days to 97 days), NT (nuchal translucency) is a fluid-filled subcutaneous space at the fetal back neck. Between 11 and 14 weeks of pregnancy, the measurement of fetal NT offers an accurate result for chromosomal abnormalities. NT thickening has been linked to a variety of defects, including aneuploidy, non-aneuploidy, fetal death, and intrauterine infections [5]. NT thickness is increased in about 75% of trisomy 21 babies, and the nasal bone is missing in 70% of the fetuses.

Babies born with Down’s syndrome (also called trisomy 21) may have underdeveloped or nonexistent nasal bones, resulting in a flat bridge. Nasal bones are present in babies with Down’s syndrome, but they are either too tiny to show on a scan or the bridge of their noses is flat. One study showed that forty-three of fifty-nine (73%) babies with trisomy 21 lacked a nasal bone, whereas just three of six hundred and three (0.5%) fetuses with normal chromosomes did so. In cases where the nasal bone is lacking, the risk of having trisomy 21 is 1.46 (95% CI 50–434), whereas in cases where the nasal bone is present, it is 0.27 (95% CI 0.18–0.40) [6].

The adverse fetal risks, such as chromosomal and other defects, as well as fetal and postnatal mortality, may be accurately predicted using fetal NT thickness. The current research shows that prenatal chromosomal abnormalities may be identified in the first trimester by assessing the NT thickness [7]. Measurements using a population-dependent growth chart are used to determine abnormal and normal baby growth. Inter- and intra-clinician heterogeneity exists in manual measurements of fetal parameters. Automatic fetal parameter measuring techniques eliminate inconsistency and provide more precise and repeatable results [8]. Automated fetal monitoring enhances workflow efficiency by allowing doctors to assess fetal data more efficiently and make better estimates based on them. One of the most sophisticated methodologies for effective prenatal identification has been found as machine learning (ML) [9].

### Contributions of This Research

⮚ We obtained fetus photographs from Boston Children’s Hospital by hand. A total of 100 fetal ultrasound images were collected and analyzed for abnormalities.
⮚ The image is then improved using hybrid maxpool matrix histogram analysis after being preprocessed with a linear contour size filter.
⮚ To obtain high-quality image segmentations, we combine a linear contour filter detector with a two-step generic grouping algorithm.
⮚ In this study, we describe how the Adaptive Stochastic Gradient Descent Algorithm was utilized to quantify risk of the incorrect categorization of fetal nuchal translucency.

This research focuses on fetal nuchal translucency aberrant risk classification using the Adaptive Stochastic Gradient Descent Algorithm, which is used to assess the risk of fetal abnormalities. Section 2, covers the literature review. Section 3 outlines materials and methods. In Section 4, the suggested method’s performance is evaluated. Section 5 presents the conclusions and future work.

## 2. Related Works

The following are some of the research papers which focus on fetal abnormality. In [10], the authors suggested that prenatal ultrasonography is an important medical indicator for aneuploidy screening, diagnosing congenital anomalies in the fetus, and monitoring the growth of the fetus. In high-risk pregnancies, ultrasound examination accuracy is reduced when the mother is obese. Prenatal ultrasound on people who are obese can be problematic, and this study seeks to provide proof and practical advice on how to treat these individuals in the ultrasound room. In [11], the authors mentioned the women’s experiences with fetal alcohol syndrome abnormality discovered during a standard pregnancy ultrasound. Their study was to learn about women’s encounters with caregivers after a fetal abnormality was discovered during a regular second-trimester ultrasound screening. In [12], the authors mentioned that third-trimester scans are largely utilized to avoid unfavorable outcomes connected to fetal growth abnormalities. Unexpected fetal abnormalities are rarely discovered from the growth scans of the third trimester. The incidence and kind of fetal abnormalities discovered in females undergoing a regular third-trimester growth scan are investigated in this study. In [13], the authors discussed the prevalence of autopsies following a fetal abnormality termination and how it affects future counseling. In recent decades, fetal autopsy after pregnancy interruption for prenatally identified fetal malformations has been used to investigate the relevance of a neonatal autopsy in an era of improved early diagnostic tools. In [14], the authors’ goal was to give a framework for physicians using the SPIKES approach; this acronym stands for setting, perception, invitation, knowledge, empathize, summary, and strategy. The SPIKES procedure concerns delivering bad news about an abnormal pregnancy to pregnant mothers. In [15] the authors proposed a unique convolutional neural network-based method for automatically detecting 13 fetal standard views in freehand 2-D ultrasound images and providing fetal structural localization through a bounding box. The network learns to locate the target anatomy dependent on image-level labels alone with weak supervision, which is a big contribution. While offering the best possible outcomes, the network structure is meant to function in real-time. The suggested technique in [16] uses deep Convolutional Neural Network (CNN) frameworks to detect the region of interest (ROI) of the fetal biometrics and organs region in the US images. This technique takes into account both regular and aberrant US data. In addition, the neural network’s input sources are supplemented with local phase information in addition to the original US data. The additional input information aids in the improvement of several neural networks’ performance. It is becoming more frequent, as in to find structural problems in embryos and fetuses as early as the first trimester. The difference between identification in the early weeks, when the embryo or early fetus is small and transvaginal ultrasound is employed, and identification in the late weeks, when NT screening, which is commonly done with transabdominal ultrasound, is critical. Early first-trimester anomalies are frequently diagnosed by chance based on clinical signs and symptoms, whereas late first-trimester diagnoses are the result of rigorous ultrasound marker screening [17]. In [18], the author discussed that when faced with a prenatal analysis of fetal abnormalities, women are faced with emotionally difficult decision-making alternatives and a great deal of uncertainty about the implications of their choices on their families. Prenatal fetal abnormality diagnosis leaves childbearing women with tough options, including continuing the pregnancy without intervention, abortion, and, in some situations, experimental fetal therapy. It is a time of transition, marked by pain and loss, regardless of their decision. Women’s and families’ experiences with the choice they make have an impact on their short- and long-term well-being. Healthcare practitioners have a critical role in easing this transition and supporting family needs. In [19] the authors’ goal was to (1) compare the outcomes of 2D ultrasound and 3D ultrasound examinations in babies with known defects, (2) assess what new information was acquired from the 3D ultrasound technology, and (3) establish the clinical significance of the 3D ultrasound approach. In [20] the authors suggested that the availability of multiple presentation modes and standardized testing allows both normal and pathological fetal anatomy to be demonstrated in regulated planes and rendered images from various perspectives. Even minor prenatal anomalies may be portrayed in a finely rendered surface or transparent picture seen from the appropriate viewpoint on an ideal sectional plane. When counseling parents, the photographs might assist them to understand the seriousness of an existing deformity or, conversely, persuade them that there are no fetal abnormalities.

Ref. [21] focused on the bagging ensemble ML technique for identifying fetal well-being by classifying fetal heart rate data as abnormal or healthy. Using antepartum cardiotocography (CTG) information, they examined the performance of the ensemble ML classifiers. Ref. [22] looked at the viability of utilizing deep-learning algorithms to identify sonographic pictures of the embryonic brain taken in typical axial planes as normal or anomalous. The training data was utilized to train the algorithms for three different aspects, namely picture segmentation along with the fetal skull, image categorization as normal or anomalous, and lesion location. Ref. [23] used normal clinical T2-weighted MR fetal brain scans to create an attention-dependent deep residual network for estimation of fetal brain age. Variations from the typical trajectories are indicators of brain anomalies, and MRI-dependent assessment of brain age is frequently utilized to evaluate ordinary brain growth. Using an ensemble technique, the network’s prediction uncertainty and estimate confidence were concurrently assessed as indicators for identifying fetal brain malformations.

Ref. [24] used classification techniques based on computational intelligence to evaluate the probability of fetal trisomy 21 and other chromosomal defects at 11–13 weeks of pregnancy. An artificial neural network was trained using a training dataset. Then, to stratify risk and diagnose instances of aneuploidy in the dataset, a two-stage technique was adopted. Pregnancies in the data group were divided into risk and no risk groups in Stage 1, utilizing four markers. Pregnancies that were not in danger were not evaluated further, but those that were at risk were referred to Stage 2 for additional assessment. Pregnancies were categorized into 3 levels of risk such as no risk, medium risk, and high risk in Stage 2, utilizing seven markers. Ref. [25] developed a deep learning system that learns, estimates, and validates fetal parameters by applying five CNN designs in a certain sequence. The CNN’s layers extract and categorize the various characteristics in US pictures automatically, and the network outputs a result. The CNN’s enhanced number of hidden layers allows it to extract even the pictures’ hidden characteristics. The collected characteristics are automatically categorized, which improves the accuracy of illness identification using fetal ultrasound pictures. Ref. [26] created a fetal health prediction system with assistive e-Health apps that can be used by both pregnant women and practitioners. They examined the performance of nine binary classification models for fetal abnormality prediction namely Bayes Point Machine, Averaged Perceptron, Support Vector Machine, Decision Jungle, Decision Forest, Logistic Regression, Locally-Deep Support Vector Machine, Boosted Decision Tree, and Neural Network. Refs. [27,28] proposed an automated NT identification approach in the NT midsagittal plane depending on a scale-invariant feature transform (SIFT) key point and a general regression neural network (GRNN). This non-invasive method is critical not just for NT testing, but also for detecting severe abnormalities and identifying high-risk pregnancies. The authors of [29,30] offer a unique paradigm for prenatal brain categorization at an early age. Diagonal quadratic discriminates analysis (DQDA), K-nearest neighbor (K-NN), random forest (RF), naive bayes (NB), and radial basis function (RBF) neural network classifiers are among the machine learning classifiers used in the research. Furthermore, employing RF, NB, and RBF network classifiers, many bagging and Adaboosting ensembles frameworks were built. Such ensembles’ results were contrasted with their separate models. The findings indicate that the innovative technique may effectively detect and categorize a variety of abnormalities in infant brain MRI pictures from a variety of GAs. The goal of this research [31] is to see how often ultrasonography results influence clinical care on the day of blood test for cell-free DNA (cfDNA) analysis. The current data in this study from the fetal medicine unit is being used to detect limitations in prenatal diagnostic techniques used in the primary obstetric care individuals in coastal Karnataka [32]. The authors examined the literature to see at what gestational age the anomaly must nearly always be identified. The gestational age when each of these disorders was identified and reported was recorded using this as a baseline. They were contrasted and analyzed to determine the effectiveness of prenatal analysis in the referral group. To examine the influence on prenatal death and morbidity, the ultimate perinatal outcome was also recorded. Ref. [33] describes a new approach for direct fetal abnormality categorization in head ultrasound pictures, allowing for quantitative evaluation of healthy or fetal hydrocephalus instances. The suggested technique is concerned with the extraction of discriminant textural characteristics from a fetal head dataset, which may aid in the detection of brain disorders. The fundamental contribution of this paper is the introduction of a completely automated technique to prenatal hydrocephalus identification based on significant textural aspects, intending to assess its ability to evaluate aberrant patients in less time. A short description of the clinical signs that may hint at a heart problem is presented in [34]. Following that, a critical examination of the efficiency of prenatal and neonatal pulse oximetry analysis is conducted, with a focus on lesions that may be overlooked. There are suggestions about how to increase diagnosis accuracy. The efficacy of extended carrier screening in assessing heritable causes of congenital abnormalities discovered by prenatal ultrasonography was investigated in the publication [35]. A retrospective record review was carried out to gather structural abnormalities and genetic testing data on newborns that were assessed by a medical geneticist postnatally. These were utilized to see whether pre-delivery extended carrier screening may have identified the cause. Furthermore, recessive and X-linked disorders on clinically accessible carrier screening panels were examined to assess the number of conditions connected to aberrant ultrasound results. The authors of [36] investigated whether postnatal neuroimaging beyond 6 months affects the diagnostic accuracy of in utero magnetic resonance imaging (MRI) and its capacity to predict the developmental outcome. Participants’ development was classified as normal, at risk, or abnormal by a pediatric neurologist and neonatologist, and the effectiveness of iuMRI and ultrasonography to predict developmental outcomes was tested. The researchers looked at the effectiveness of regular ultrasound examinations at 35–37 weeks of pregnancy in diagnosing previously undiscovered fetal abnormalities [37]. The nature and occurrence of new abnormalities were established, and anomalies were categorized according to the afflicted main organ system. In [38], the diagnostic utility of 4D ultrasonography coupled with the spatio-temporal image correlation (STIC) approach for fetal heart malformation and chromosomal disorders in early pregnancy was explored. Ref. [39] proposes using statistical characteristics and pattern classifiers such as support vector machines to automate CHD identification from ultrasonic 2D images.

### Problem Statement

Earlier prediction of fetal abnormalities during prenatal screening would make treatment therapies more accessible to minimize infant morbidity and death. The detection of NT and measurement of thickness is a significant marker in the identification of fetal abnormalities. Manual estimation of risk associated with fetal anomalies based on NT thickness is quite time-consuming and inefficient. Deep learning approaches have recently been used for automated NT evaluation, diagnosis of serious abnormalities, and identification of high-risk pregnancies. Though existing techniques showed better accuracy in evaluating the fetal risks, enhancements must be carried out to improve the efficiency. Here, we applied a novel Adaptive Stochastic Gradient Descent Algorithm (ASGDA) approach for classifying fetal abnormalities into high and low-risk cases.

## 3. Materials and Methods

Fetal photos were collected from Boston Children’s Hospital in previous work. A total of 100 fetal ultrasound photos were evaluated for anomalies that include multiple pregnancy of few women. The image is then preprocessed with a linear contour size filter before being enhanced with hybrid maxpool matrix histogram analysis.

### 3.1. Linear Contour Size Filter

A linear contour filter detector with a two-step generic grouping technique is used to achieve high-quality image segmentations. The Focused Watershed Change, an image file modification for constructing a series of beginning areas with an angled contour input, is described initially. Next, an unsupervised clustering approach is used to build a hierarchy from those sections, which is depicted by Ultrametric Contour Lines, a real-valued image formed by valuing every frontier by its degree of vanishing. User-specified labels can be used to further improve these multilayer segmentations. A linear contour size filter has the advantage that representing the presence of genuine fundamental contours is quite simple, by linking a binary random variable to them. The Oriented Watershed Transform (OWT) assigned boundary strength to an arc, which can be interpreted as an approximation of the chance of that curve having an accurate contour. Uncertainty about segmentation is not easily represented.

The Ultrametric Contour Map (UCM) is one alternative that defines a duality between closed, non-self-intersecting weighted contours and a hierarchy of regions. This hierarchy’s base level preserves even weak contours, over segmenting the image. As a result of this, the hierarchy is under-segmented. Moving between levels involves a constant trade-off. This change in representation allows later processing stages to use information from various levels or select a level based on additional knowledge.

### 3.2. Hybrid Maxpool Matrix Histogram Analysis

**Figure 2:**
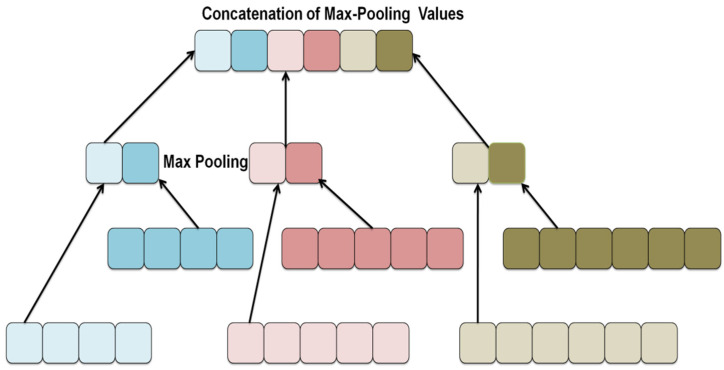
Framework of Hybrid Maxpool matrix.

Most prior stochastic pooled techniques use a theory to predict a pooling strategy for every pooling region. Unlike certain techniques, hybrid max-pooling chooses a pooling technique for every convolution layer based on probabilities, now with general max-pooling facilities for all pooling areas. The framework of a hybrid maximum pool is depicted in Figure 2.

The approach was influenced by the classification device’s dropping process. The dropout keeps certain networks passive with a preset chance within every test stage, or the outcomes of the dormant nodes are reduced to 0. As a consequence, the program is required on a wide range of alternative systems, overcoming the risk of overfitting.

In the testing process, the average of the outputs for those possible systems is conducted to identify a node’s result. Furthermore, our pooling approach is motivated by the belief that retraining the system with a variety of descriptors and then combining them during the trial phase will boost generalization. For those sharing areas in the learning phase one per dataset in the convolutional layer, we use averaged pooling with frequency r and max-pooling concerning frequency r. As a consequence, the pooling approach with a convolutional feature map is superior and yields the following pooling feature map:

(1) $$G=\left\{\begin{array}{c} G_{ave}\left(withprobabilityr\right),\;\;\;\;\;\;\;\;\;\\ G_{max}\left(withprobability\;1-r\right), \end{array}\right.$$

where *G_ave_ = {* $G_{1_{ave}}$, *…*, $G_{J_{ave}}$ *}* with $G_{J_{ave}}$ *= (1/|A_b_|) b∈A_b_* *a_i_* and *G_max_ = {G_max1_*, *…*, $G_{max_{b}}$*}* with $G_{max_{b}}$ *= max_1_∈* *A_b_* *a_i_.*

For each pooling region, the predicted measured value in Equation (1) below is used as the output.

(2) $$G=G_{hyb}\equiv rG_{ave}+\left(1-r\right)G_{max}$$

Figure 2 shows a schematic representation of hybrid pooling. We may build a variety of CNN models by stochastically mixing the 2pooling algorithms for multiple feature maps, while also examining the influence of every convolutional layer and the features of the treated image data. Equation (2) represents the median of such various models. If *r* = 0, hybridization pooling corresponds to maximal pooling, so when *r* = 1, it corresponds to the pooling layer. In composite pooling, the chance parameter values can be selected layer by layer.

**Figure 3:**
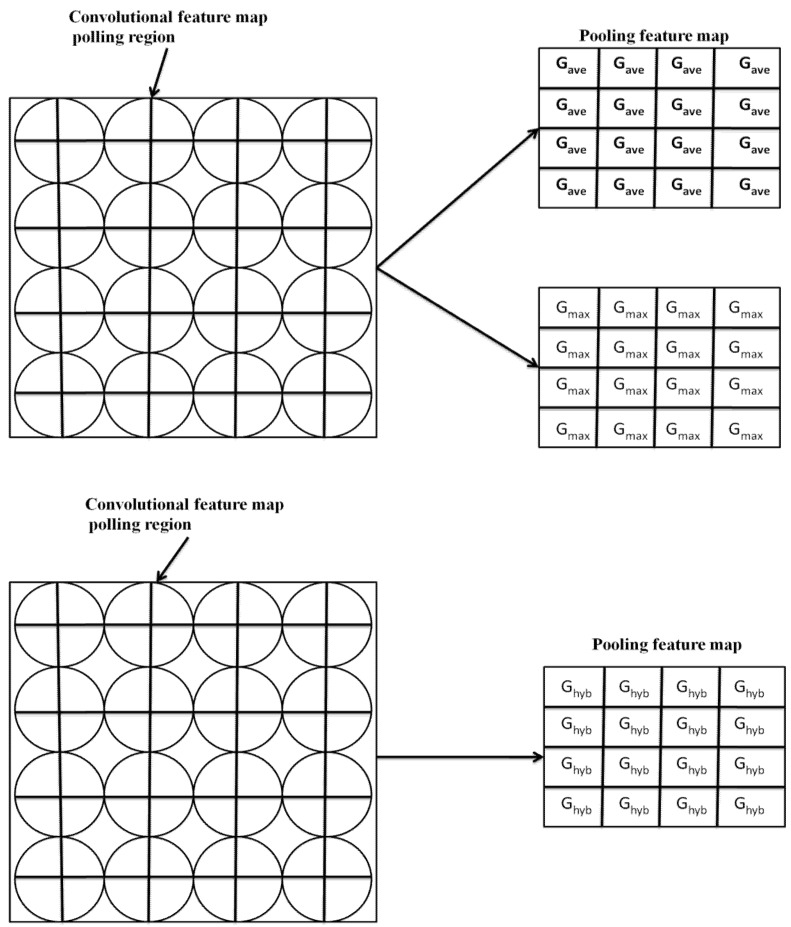
Convolutional hybrid maxpooling.

Figure 3 depicts the schematic illustration of hybrid max-pooling. The operation is the training stage. *G_ave_* and *G_max_* are defined in Equation (1) and the operation in the testing stage *G_hyb_* is defined in Equation (2).

Now, in this paper, we will briefly discuss how the Adaptive Stochastic Gradient Descent Algorithm was used to assess risk in the classification of aberrant fetal nuchal translucency. Many studies focus on the risk of Down’s syndrome; however, in our study, we briefly discuss nuchal translucency thickness, which can be linked to a variety of defects, including:

⮚ Aneuploidy
  - Turner syndrome
  - Trisomies

⮚ Non-aneuploidy
  - Noonan syndrome: the only genetic molecular disorder that has been linked to increased nuchal translucency
  - congenital heart disease (3.5 mm): the risk ranges from 2 percent at the 95th percentile to 5 percent at the 99th percentile, with septal abnormalities being the most prevalent anomaly
  - omphalocele
  - congenital diaphragmatic herniation
  - Smith-lemli-Opitz syndrome
  - VACTERL association
  - skeletal dysplasias
⮚ Fetal death: the risk is proportional to nuchal translucency increased thickness, ranging from 1.6 percent for nuchal translucency between the 95th and 99th percentiles to 20% for NT > 6.5 mm
⮚ Intrauterine infections: apathogen with a direct relationship to increased nuchal translucency is parvovirus B19, which is influenced by fetal anemia and fetal cardiac infarction.

**Figure 4:**
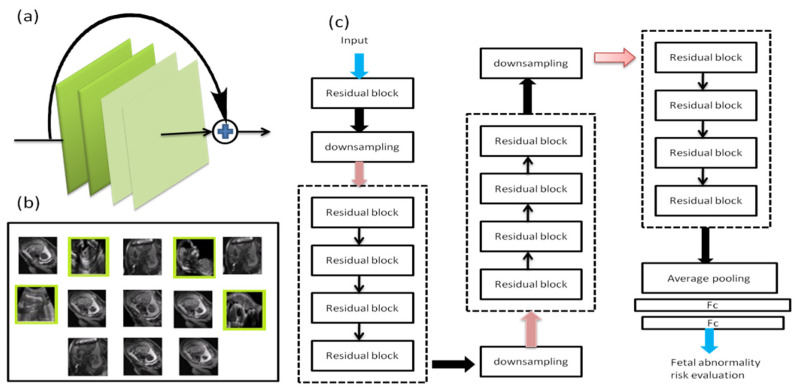
Schematic diagram of basic network architecture. (**a**) The bottleneck blocks, (**b**) The network’s input types, (**c**) Basic architecture of the network.

The bottleneck block (Figure 1) was selected as the essential residual block in this study. It was made up of a batch normalization (BN) convolution layer and a rectified linear unit (*ReLU*). The basic network designs are represented in Figure 4.

Before sending specific input data onto the next layer in the network, BN layers are utilized to normalize its activations. The BN layer is very good at cutting down on the number of epochs required to train a neural network. It also helps to stabilize training by allowing for a greater diversity of learning rates and regularization strengths. The execution of BN is explained below. The mean, *μ_v_*, and variance, *σ_v_*^2^, of the mini-batch mean are calculated from Equations (3) and (4).

(3) $$\mu_{V}=\frac{1}{N}\overset{N}{\underset{n=1}{\sum }}s_{n}$$

(4) $$\sigma_{v}^{2}=\frac{1}{N}\overset{N}{\underset{n=1}{\sum }}{\left(s_{n}-\mu_{v}\right)}^{2}$$

where *S* denotes the input and *n* = 1, 2, ……, *N*. The normalized value of the input *S_n_* is computed using Equation (5).

(5) $$s^{\prime }{}_{n}=\frac{s_{n}-\mu_{V}}{\sqrt{\sigma_{V}^{2}+\varepsilon }}$$

where *S′_n_* is the normalized input value and ε means the smaller positive value to avoid the divisor as zero. The output image *w* after BN is defined by Equation (6).

(6) $$w_{n}=\gamma s^{\prime }{}_{n}+\beta$$

where *γ* and *β* denote the two parameters that are learned through backpropagation.

Figure 4. (a) The bottleneck blocks, (b) The network’s input types, (c) Basic architecture of the network. The network consists of 3 layers of bottleneck blocks, each followed by stride 2 downsampling residual blocks.

The approach uses three stochastic modules, each with a Mask Branch (MB) and Trunk Branch (TB). In the first and second modules, the TB was constructed of several residual blocks to produce features of the aberrant prenatal image. On Forward implications, the MB chose features of the aberrant fetus image.

As a result of the residual block following the max-pooling stage, feature maps (low resolution) with improved semantic information were generated. Then, bilinear interpolation was used to transfer the low-resolution features into high-resolution features using the asymmetric top-down framework. Before the final convolutional layer (CL), each channel feature map was normalized using a sigmoid function. After each CL and fully-connected layer, non-linear activation functions are introduced. Otherwise, the whole system remains linear, and the nonlinearity in the input picture cannot be mapped to the target labels. The sigmoid function has traditionally been utilized as a nonlinear activation function and is defined by Equation (7).

(7) $$D_{1}\left(s\right)=\frac{1}{1+\exp\left(-s\right)}$$

where *s* is the input and *D(s)* refers to the sigmoid function.

In the realm of deep learning, the rectified linear unit (*ReLU*) is quickly gaining popularity. It is employed to replace the conventional sigmoid function because they are associated with information loss and a larger computational amount for generating error gradients. *ReLU* is described by Equation (8).

(8) $$ReLU\left(s\right)=\left\{\begin{array}{l} \begin{array}{ll} \;\;s & if\;x>0 \end{array}\\ \begin{array}{ll} 0 & otherwise \end{array} \end{array}\right.$$

The amount of computing required for the whole process is substantially less using the *ReLU* activation function. Furthermore, *ReLU* will render the output of certain neurons zero, resulting in network sparsity and reduced parameter dependency. As a result, *ReLU* will solve the problem of over-fitting. In addition, *ReLU* accelerates the convergence of ASGDA, which is another advantage.

**Figure 5:**
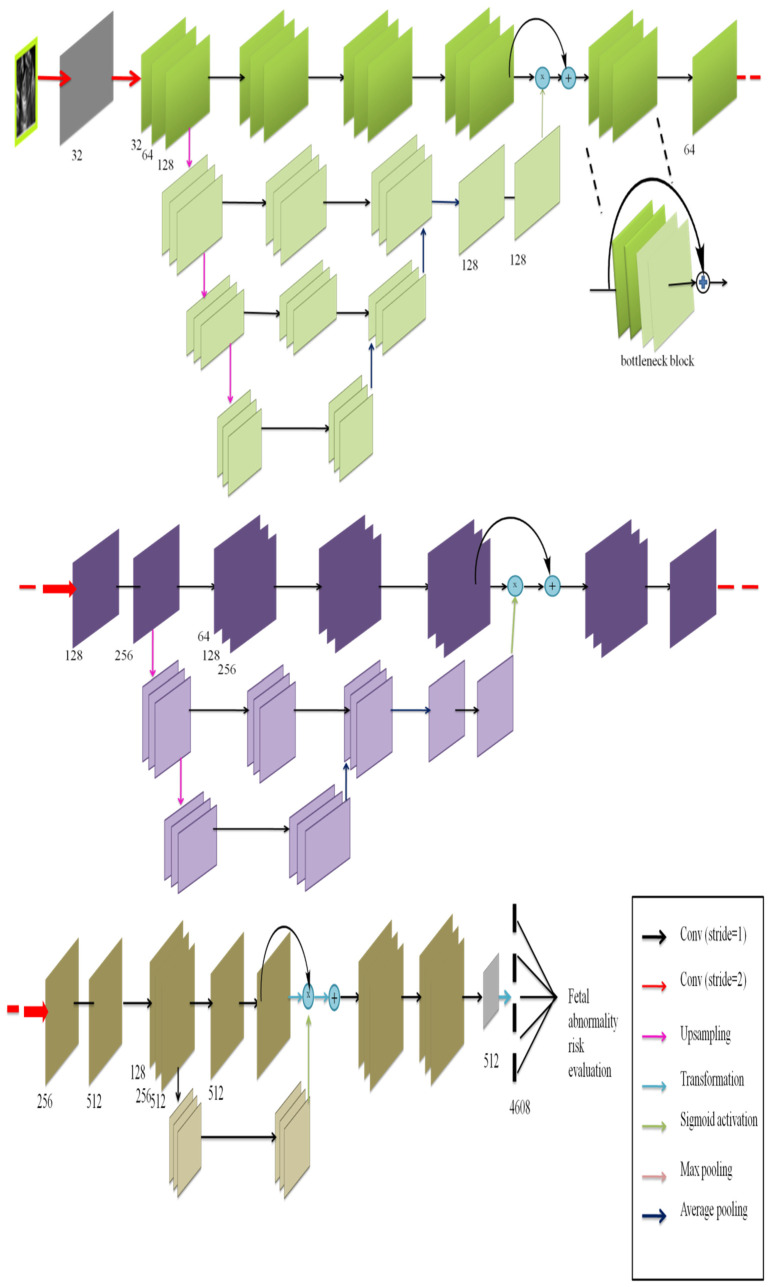
Illustration of the proposed network.

As indicated in Figure 5, there are three stochastic modules. Each module is divided into two parts: a TB and an MB. The layer the number of channels available is indicated, and in the stochastic module, all the residual blocks are constructed using the same design.

The two branches were united by multiplying the two branches by the TB forgo connection. Leftover blocks between the bottom-up and top-down structures were combined, information from multiple scales to be integrated into a single structure. The bottom-up and top-down structures were removed in the third module, and a sigmoid function followed by a mask was built at a higher layer to promote semantic abstraction. To maintain the same features as the second stochastic module, the TB only preserved convolutional layers. Finally, average pooling was used to estimate the risk score of fetal anomalies in consecutive residual blocks, preceded by a fully-connected layer. Overfitting and computational load may occur if the activation map includes too many features. To achieve reduction, a pooling layer, which is a kind of nonlinear subsampling, is often utilized. We applied two pooling layers in our experiments such as max and average pooling. The average pooling method selects the components’ average value in each pooling zone, while the maximum pooling method selects the highest value.

The activation set ‘*T*’ included in pooling region ‘*X*’ was defined by Equation (9).

(9) $$T=\left\{t_{h}\left|h\in X\right.\right\}$$

The average pooling (*LT*) was defined by Equation (10).

(10) $$LT=\frac{\sum T_{X}}{\left|T_{X}\right|}$$

The max pooling (*NT*) was defined by Equation (11).

(11) $$NT=\max\left(T_{X}\right)$$

This research looked at two different sorts of maps. The stochastic activation map was produced from the output maps of each stochastic module, and mask weight encoding spatial information was created in each stochastic module. This demonstrated the role of location in risk prediction. Concerning the fetal image, saliency maps were computed, revealing the regions that could have a significant impact on the risk prediction of fetal abnormalities. To avoid the backward flow of negative gradients, guided backpropagation was used in the calculation, which removed noisy patterns in the saliency maps.

### 3.3. Uncertainty Estimation

Aleatoric and epistemic uncertainty makes up the predicted uncertainty in a probabilistic model. The ambiguity in the result (fetal abnormality risk b) for a given image is captured by the aleatoric uncertainty information. The variance of the conditional distribution *p*(*b|a,w*), where w specifies parameter weights on dataset D, is used to estimate it. A heteroscedastic Gaussian distribution is used to model the uncertainty.

(12) $$p\left(b|a,w\right)=N\left(b;\mu \left(a,w\right),\sigma^{2}\left(a,w\right)\right)$$

where $\mu \left(a,w\right)$ is the expected risk, variance is $\sigma^{2}\left(a,w\right)$ and $\delta_{x}\left(b\right)$ is the calculated aleatoric uncertainty. During training, the loss function was the negative log-likelihood (NLL), and $\sigma^{2}\left(a,w\right)$ was acquired.

A collection of ensembles is used to estimate epistemic uncertainty (model uncertainty). With random initialization of model parameters on a shuffled training set, the network was trained L times with different initial conditions of model parameters on the training set. As a result, L networks with various specifications ${\left\{w\right\}}^{L}{}_{l=1}$ could be created.

The variance of anticipated risk was used to calculate epistemic uncertainty $\delta_{e}$. The mean of the ensembles could be used to assess the expected fetal risk. The calculated aleatoric and epistemic uncertainty of the network ensembles, which together made up the final predictive uncertainty $\delta^{2}\left(b\right)=\delta_{x}^{2}\left(b\right)+\delta_{e}^{2}\left(b\right)$, were calculated using Equations (14) and (15).

(13) $$\hat{\mu }\left(a\right)=\frac{1}{L}\underset{l}{\sum }\mu \left(a,w_{l}\right)$$

(14) $${\hat{\delta }}^{2}{}_{x}\left(b\right)=\frac{1}{L}\underset{l}{\sum }\sigma^{2}\left(a,w_{l}\right)$$

(15) $${\hat{\delta }}^{2}{}_{x}\left(b\right)=\frac{1}{L}\underset{l}{\sum }\mu^{2}\left(a,w_{l}\right)-{\hat{\mu }}^{2}\left(a\right)$$

In a 2D network architecture, the scales $\hat{\delta }$ varied among slices, and each slice had its pattern, resulting in variable parameters. To mention this, we proposed the anticipated confidence risk *C*(*a,b*).

(16) $$C\left(a,b\right)=N\left(b;\hat{\mu }\left(a\right),\hat{\delta }\left(b\right)\right)=\frac{1}{\sqrt{2\pi }\hat{\delta }\left(b\right)}e^{-\frac{{\left(y-\hat{\mu }(a)\right)}^{2}}{2\hat{\delta }{\;}^{2}\left(b\right)}}$$

The risk of fetal abnormality is predicted using the Adaptive Stochastic Gradient Descent Algorithm. This is an optimization approach for determining the model parameters that best match projected and actual results. It is a very effective strategy. Finding model parameter values that are compatible with previous knowledge and produce the greatest fit with the least prediction error with observed data is an optimization issue in the data fitting of this experiment. ASGDA is a method for minimizing an objective function that is characterized by the parameters of a model by updating the parameters in the opposite direction of the objective function’s gradient. ASGDA works with a single training dataset at a time and then adjusts the weights for each row of data iteratively. To improve the performance of deep convolutional neural networks, ASGDA is used. It aids a model’s training during backpropagation when weights are modified to reduce loss error. Equation (17) defines the convolution operation as performing a weighted average operation at each time interval.

(17) $$d\left(i\right)=\int s\left(l\right)a\left(i-l\right)ol=o\left(t\right)=\left(s*a\right)\left(i\right)$$

where ‘*s*’ means the input and ‘*a’* denote the filter or kernel, and ‘*o*’ defines the feature map which is the output for continuous-time series ‘*i’*.

By traveling along the direction of the steepest gradient determined using random selections of the data, the ASGDA minimizes the loss error function (j) according to Equation (18).

(18) $$\theta_{i+1}=\theta_{i}-\eta i\nabla_{\theta }\left(\frac{1}{\left|B_{t}\right|}\underset{j\epsilon B_{i}}{\sum }J_{j}\left(S_{j},\theta \right)+Y\left(\theta \right)\right)$$

where θ refers to the model parameter, ηi defines the learning rate, Y means the regularizing function, and B_t_ defines the training data batch that contains randomly selected data.

Thus, ASGDA helps in predicting the risk associated with fetal anomalies with minimum prediction error.

Algorithm 1 depicts the Adaptive Stochastic Gradient Descent. Using ASGDA, it is possible to forecast the likelihood of fetal abnormalities with the smallest amount of error. Performance evaluation includes sensitivity, Matthias Correlation Coefficient (*MCC*), accuracy, precision, recall and F1-score.
**Algorithm 1:** Adaptive Stochastic Gradient Descent AlgorithmInput: Fetal abnormality images.Output: Risk prediction start: # Remove the noise in the fetal abnormality imagesDo training data classification   for j = 1 to Datainitnumpy (array) → size       [A,B,C]         returns: [(class image)]       returns: [corres, m(images)]     end forpat_size = array(array, shape)  class = trans (gauss_noise)  m = trans (spat)  m = m [0]       return (class [0], m)    for each i in data returns: sample(w)n_samples = l(s_labels)CountDiC = dict(unique, Count)     w = [ ]     for each label in s_labels        Append (w) → (n_samples/count labels)        return w       end for    end for   update w   while (iter<= imagei)End

As an analogy, think of a medical diagnostic test. The test can correctly identify patients who are ill with the condition that is being tested for. The sensitivity (also known as the detection rate in a clinical setting) of a test used to identify a condition is the percentage of people who test positive for the disease among those who have it. This can be expressed mathematically in the form of the Equation (19)

(19) $$Sensitivity=\frac{numberof\;t_{p}}{numberof\;\;t_{p}+f_{n}}$$

A contingency matrix method of calculating the Pearson product-moment correlation coefficient between actual and predicted values is the Matthews correlation coefficient. MCC’s entries are summarized as follows:

(20) $$MCC=\frac{\left(t_{p}*t_{n}-f_{p}*f_{n}\right)}{\sqrt{\left(t_{p}+f_{p}\right)\left(t_{p}+f_{n}\right)\left(t_{n}+f_{p}\right)\left(t_{n}+f_{n}\right)}}\;$$

There is a method for determining how many samples can be successfully categorized. It decides how near the findings are to the expected result. It is obtained by dividing the sum of genuine positives and true negatives by the sum of anticipated positives and negatives.

(21) $$Accuracy=\frac{t_{p}+t_{n}}{t_{p}+t_{n}+f_{p}+f_{n}}\;$$

The number of correctly predicted favorable events is measured by precision. A percentage of all successfully projected positive examples is used to calculate a correctly anticipated positive example. In other words, the true positive is produced by dividing the sum of true and positive.

(22) $$Precision=\frac{t_{p}}{t_{p}+f_{p}}\;$$

It can quantify the recall rate by dividing the number of true positives by the total number of true positives and false negatives in a sample of data.

(23) $$Recall=\frac{t_{p}}{t_{p}+f_{n}}\;$$

The F1 score is obtained as the harmonic mean of precision and recall during a certain period. It is a statistical metric for evaluating performance. As a result, both s and false negatives are factored into this score.

(24) $$F1-score=\frac{2\times precision\times recall}{precision+reccall}\;$$

## 4. Results

The adaptive stochastic gradient descent technique predicts the risk rate for fetal abnormalities, which is proportional to nuchal translucency thickness:

- It has a risk of 1% at 2 mm.
- It has a risk of 7% at 3.4 mm.
- It has a risk of 20% at 3.5–4.4 mm.
- It has a risk of 50% at 5.5–6.4 mm
- It has a risk of 75% at ≥8.5 mm.

The nuchal translucency thickness was less than4.5 mm in most trisomy 21 fetuses, 4.5–8.4 mm in those with trisomies 13 or 18, and 8.5 mm or greater in those with Turner syndrome.

Specifications such as (a) accuracy (b) precision (c) recall and (d) F1-score are used to validate the suggested approach’s behavior. This evaluation will consider four factors: $t_{p}$ denotes True Positive and True Negative is represented as $t_{n}$. False Positive and False Negative are represented as $f_{p}$ and $f_{n}$.

- $t_{p}$ denotes that the risk is normal, and it turned out to be exactly that.
- $t_{n}$ denotes that the risk is expected to be high, and it is high.
- $f_{p}$ denotes that the risk is expected to be high, yet it is normal.
- $f_{n}$ denotes that the risk is expected to be normal, yet it is high.

**Figure 6:**
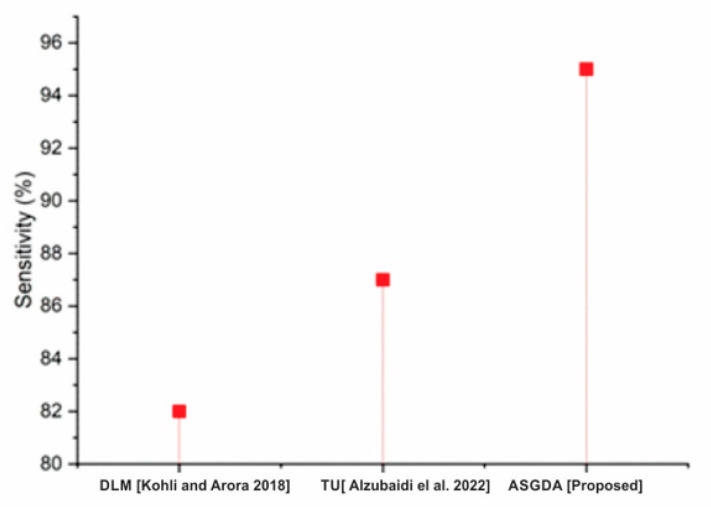
Sensitivity of proposed and existing work [40,41].

**Figure 7:**
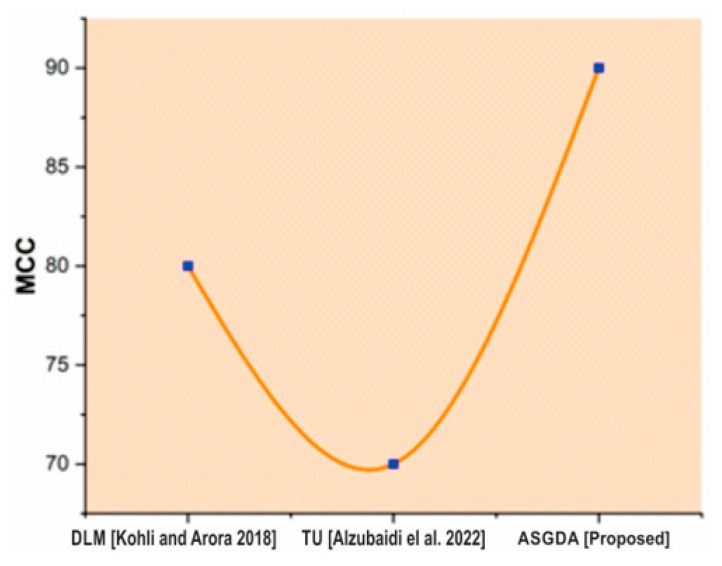
Comparison of MCC of existing and proposed method [40,41].

Different methods for finding these values are also compared to the proposed method. Figure 6 shows a sensitivity comparison graph. The proposed ASGDA work is much more advanced than two other existing works, Machine Learning Algorithm (MLA) and Artificial Intelligence (AI). The comparison graph is shown in Figure 7. It is found in MCC that the proposed work is more efficient than existing works such as Deep Learning Models (DLM) and Three-dimensional Ultrasound (TU).

**Figure 8:**
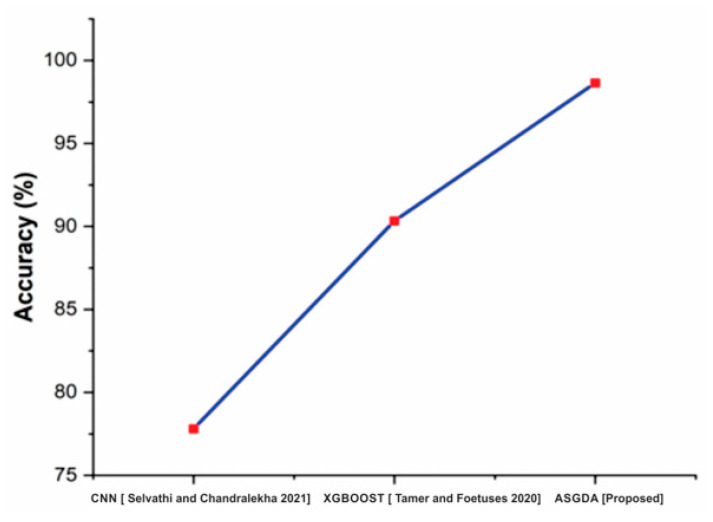
Comparison of accuracy with the traditional and proposed method [42,43].

**Figure 9:**
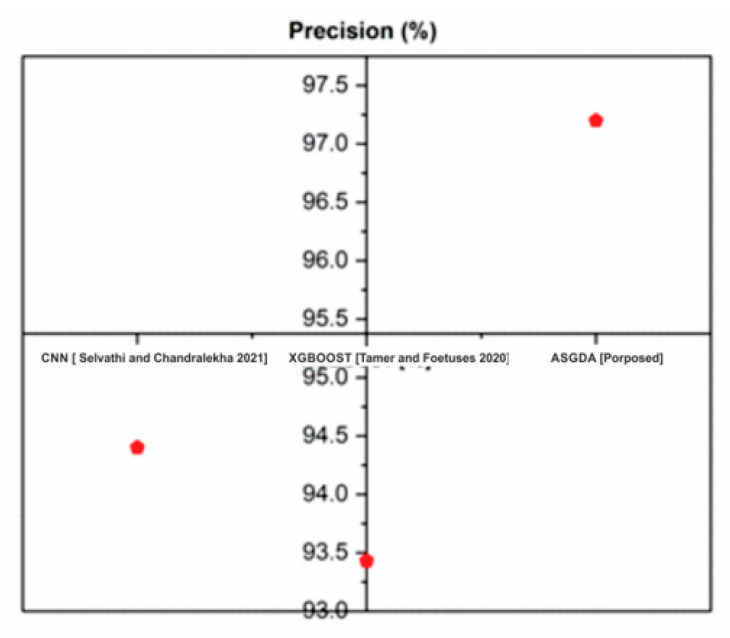
Comparison of precision with the traditional and proposed method [42,43].

Figure 8 demonstrates that the suggested method’s accuracy (98.642%) is higher than that of established approaches such as CNN (77.8%) and XGBoost (XGBoost) (90.33). Figure 9 shows that the accuracy of the proposed method (98.642%) is higher when it is compared with the traditional methods such as CNN (77.8%) and XGBoost (90.33).

**Figure 10:**
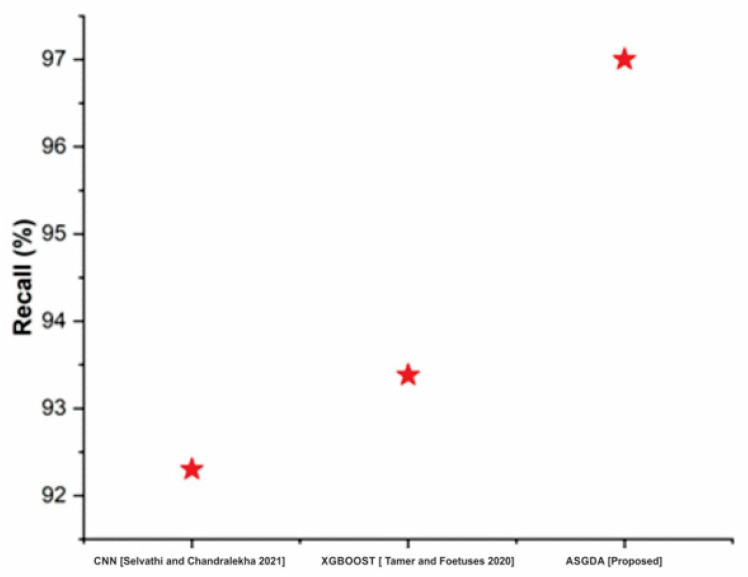
Comparison of recall with the traditional and proposed method [42,43].

**Figure 11:**
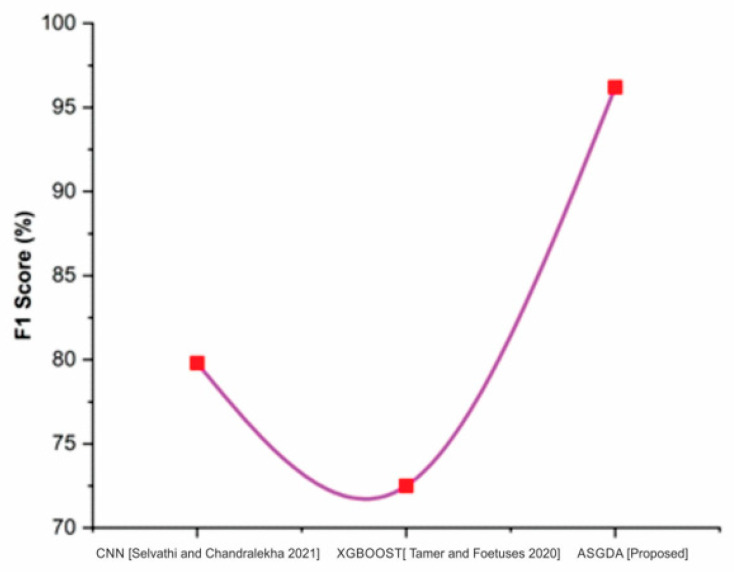
Comparison of F1-score with the traditional and proposed method [42,43].

Figure 10 shows that the recall of the proposed method (97%) is higher when it is compared with the traditional methods such as CNN (92.3%) and XGBoost (93.38%). Figure 11 indicates that the suggested method’s F1-score (96.2%) is greater than existing methods like CNN (79.8%) and XGBoost (92.5 percent). Table 1 compares CNN and XGBOOST to the proposed approaches.

**Figure 12:**
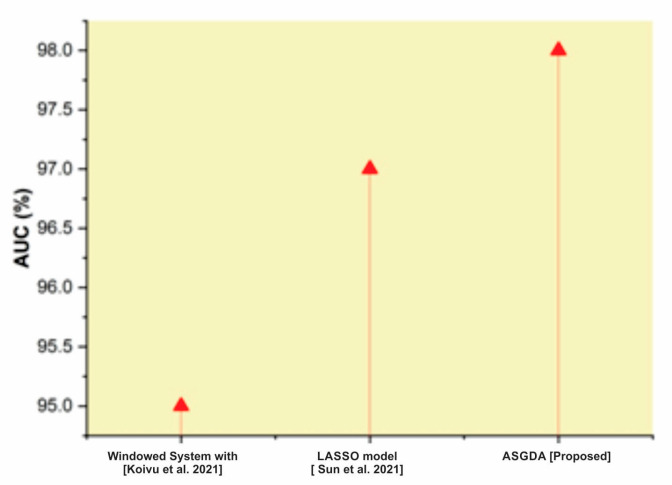
Comparison of AUC with the traditional and proposed method [44,45].

Figure 12 shows that the AUC of our proposed model was higher than that of existing techniques such as the least absolute shrinkage and selection operator (LASSO) model and windowed system with transfer learning (TL). A greater AUC value indicates that the ASGDA model classifies the risk associated with fetal abnormalities based on NT efficiently. From the result analysis, it is confirmed that the prediction performance of ASGDA model in the screening of fetal anomalies based on fetal NT is better compared to the conventional techniques.

### Discussion

Early diagnosis of nuchal translucency would enable physicians to examine at-risk fetuses more carefully before apparent characteristics emerged. This might improve perinatal outcomes and potentially lead to the discovery of additional underlying causes of illness that may otherwise have been ignored. To classify nuchal translucency at birth, we made use of machine learning models and fed those models’ data from the second trimester. We evaluated the performance of our model to the performance of a baseline clinical diagnosis, and we found that ASGDA could enhance the prediction accuracy when compared to the clinical diagnostic (Table 1) by roughly 98.642% when using the same parameters. Using AI in terms of diagnosis, complete models need to combine diagnostic imaging and clinical data. However, in practice, not all instances are straightforward, meaning that there is just one obvious deformity, and other defects may be too subtle to pick up on. The data that can be used for training cannot cover all the abnormalities that might occur in fetuses; therefore recognizing and diagnosing abnormalities based on a single aberrant image is impossible. In addition, the data that are employed often originate from the same sites, which is inadequate for improved robustness and makes the experimental data too restricted for widespread clinical applications. Because there is no radiation involved, 3D and 4D ultrasounds are just as risk-free as conventional 2D scans. Some parents are concerned, even though it is not regarded to be a danger, about the spike in temperature that is induced by the sound waves that are employed to create a picture of the baby. Deep Convolutional Neural Networks (CNN) frameworks were used to recognize the fetus biometrics and organs area of interest (ROI) in ultrasound (US) pictures. This method considers both normal and anomalous US data. Furthermore, in addition to the original US data in poor precision, the neural network’s input sources are augmented with local phase information. In the past, there have been efforts made to forecast growth-restricted babies using ultrasound images themselves. However, these attempts were unsuccessful. Because this was a retrospective study, we were unable to evaluate the inter- and intra-observer variability of the measurements. This was another one of the limitations of the study. The ultrasound measurements, on the other hand, were carried out in our hospital by highly experienced US specialists. In addition, the subjects’ identities had already been removed from the data and some of the people who participated in our research might have had more than one pregnancy. Additionally, there was a not insignificant but noticeable gap in the ages of birth between the three groups. Even if we made sure that none of the participants were born prematurely and that they did not have any other observable abnormalities outside nuchal translucency, it is still conceivable that we did not rule out all the potential underlying problems.

## 5. Conclusions

Without prenatal screening, there is a possibility that some children would be born with a condition that is aberrant relative to the usual. Because of the widespread use of ultrasounds to screen for birth defects in unborn children, prenatal care has been given much more priority in many nations. In this study, we examine the relationship between the nuchal translucency (NT) thickness level and the likelihood of fetal abnormalities. To do this, we suggest using a method called Adaptive Stochastic Gradient Descent. In a head-to-head comparison of the results obtained by the different models, the recommended technique strongly outperforms the other methods. The suggested method offers a more accurate risk estimate when compared to the alternatives; nonetheless, the superiority of the method that has been proposed demonstrates the effect of the characteristic that has been selected.

Recent research has shown that chromosomal and non-chromosomal anomalies are primarily connected with the 99th percentile value of NT thickness. Because of this, it is recommended that more studies be planned that also determine the 99th percentile values of NT thickness. It has been suggested that using these values for screening for chromosomal abnormalities during the first trimester of pregnancy is more favorable than using the recommended single cutoff value. The obtained reference range in our studied population was different from that reported for other ethnic groups. The correlation between higher NT thicknesses and chromosomal problems is another piece of evidence that supports its usefulness.

## Figures and Tables

**Table 1:** Comparison of traditional methods with the proposed method.

Algorithm	Accuracy	Precision	Recall	F1-Score
CNN [16]	77.8	94.4	92.3	79.8
XGBOOST [17]	90.33	93.43	93.38	72.5
ASGDA [Proposed]	98.642	97.2	97	96.2

## Data Availability

There are no available data to be stated.
